# Acute Kidney Injury Recognition and Management: A Review of the Literature and Current Evidence

**DOI:** 10.5539/gjhs.v8n5p120

**Published:** 2015-09-17

**Authors:** Syed Raza Shah, Sameer Altaf Tunio, Mohammad Hussham Arshad, Zorays Moazzam, Komal Noorani, Anushe Mohsin Feroze, Maham Shafquat, Huma Syed Hussain, Syed Ali Hyder Jeoffrey

**Affiliations:** 1 Department of Medicine, Dow University of Health Sciences (DUHS), Karachi, Pakistan; 2 Department of Medicine, Aga Khan University of Health Sciences, Karachi, Pakistan; 3 Department of Biological Sciences, The Lyceum School, Karachi, Pakistan; 4 Department of Biological Sciences, Bayview College, Karachi, Pakistan

**Keywords:** Acute Kidney Injury, patients, complex disorder

## Abstract

Acute renal failure is defined as a rapid decrease in the glomerular filtration rate, occurring over a period of hours to days and by the inability of the kidney to regulate fluid and electrolyte homeostasis appropriately. AKI is a catastrophic, life-threatening event in critically ill patients. AKI can be divided into pre-renal injury, intrinsic kidney disease (including vascular insults) and obstructive uropathies. The prognosis of AKI is highly dependent on the underlying cause of the injury. Children who have AKI as a component of multisystem failure have a much higher mortality rate than children with intrinsic renal disease. Treatment of AKI is subjected to risk stratification and ongoing damage control measures, such as patients with sepsis, exposure to nephrotoxic agents, ischemia, bloody diarrhea, or volume loss, could be helped by optimizing the fluid administrations, antibiotics possessing least nephrotoxic potential, blood transfusion where hemoglobin is dangerously low, limiting the use of nephrotoxic agents including radio contrast use, while maximize the nutrition. Acute kidney injury remains a complex disorder with an apparent differentiation in pathology between septic and nonseptic forms of the disease. Although more studies are still required, progress in this area has been steady over the last decade with purposeful international collaboration.

## 1. Introduction

Acute renal failure is defined as a rapid decrease in the glomerular filtration rate (gfr), occurring over a period of hours to days. This leads to an increase in serum concentration of nitrogenous excretory products and creatinine leading to the inability of the kidney to regulate electrolyte and fluid homeostasis appropriately. The term *acute renal failure* (ARF) has been replaced by the term Acute Kidney Injury (AKI), much like multisystem organ failure is now multisystem organ dysfunction, to reflect a continuum of disease, and not a single event. Furthermore, AKI affects about 7% of all hospitalized patients and around 35% of overall intensive care patients. (Cerda et al., 2008; Lattanzio & Kopyt, 2009).

Our knowledge about AKI in the recent years has increased significantly. However, despite the advances and development of efficient techniques for its treatment, mortality and morbidity rates are still very high, especially in critically ill children (Gallego et al., 2001; Lins et al., 2000). In a large study of adult patients, the incidence of AKI was around 200 patients per million population, and the most common cause of kidney injury was acute tubular necrosis in 45% of patients and pre-renal in 21% of patients (Liano & Pascual, 1996). Similar epidemiologic studies have not been performed in pediatric patients. Although a great deal of work has been published, relatively few studies have considered the prognoses of pediatric patients regarding this syndrome (Gallego et al., 2001). In a study of neonates, the incidence of AKI ranged between 8% to 24% of newborns, and AKI was particularly common in neonates who had undergone cardiac surgery (Martin-Ancel et al., 1995; Fernandez et al., 2005). Mortality rates in critically ill children with AKI are also high, ranging between 9% and 67% (Palmieri and Lavrentieva and Greenhalgh, 2009; Kendirli et al., 2007).

## 2. Epedimiology & Etiology of AKI

AKI can be divided into pre-renal injury, intrinsic kidney disease and obstructive uropathies. Some causes of Acute kidney injury, such as renal vein thrombosis and cortical necrosis, occur more commonly in neonates, whereas Heamolytic Uremic Syndrome is more common in young children, and Rapidly Progressive Glomerulonephritis (RPGN) generally occurs in older children and adolescents. Exposure to teratogenic drugs during pregnancy is one of the main causes leading to AKI in newborns interfering with embryogenic processes like nephrogenesis. These include drugs such as angiotensin receptor blockers, angiotensin-converting enzyme inhibitors and nonsteroidal anti-inflammatory drugs (Lip et al., 1997; Martinovic et al., 2001; Cooper et al., 2006; Benini et al., 2005). The history, examination, and laboratory tests such as radiographic studies and urinalysis can establish the likely cause of AKI. Often, multiple factors are likely to be implicated in the etiology of AKI such as in hospitalized children.

A study in Turkey on 100 children with Acute Kidney Injury described the most common causes as bone marrow transplantation, dehydration, renal disease, cardiac surgery and nephrotoxic medication. In yet another article from the same country, on 472 children with AKI (including 32.6% neonates), hypoxic schemic injury and sepsis were leading causes of AKI (Duzova et al., 2010; Ozeakar et al., 2009). In Kolkata, India, snake bite and glomunerulonephritis were the 2 most important causes of AKI in 37 children, making 70% of all cases (Sinha et al., 2009). In Nigeria, two different studies from two different geographic regions showed that the most common cause of AKI in children was volume depletion and that the kidney injury was due to preventable causes (Anochie & Eke, 2005; Olowu & Adelusola, 2004). Mortality rates in these studies were quite high due to scarce dialytic resources (Anochie & Eke, 2005; Olowu & Adelusola, 2004). Hence, the importance of prevention of AKI at a global level to significantly reduce mortality rates is imperative.

Other studies have demonstrated that very low birth weight, a patent ductus arteriosus, nonsteroidal anti-inflammatory drugs and maternal receipt of antibiotics and was associated with the development of AKI (Cataldi et al., 2005). Moreover, maternal ingestion of nonsteroidal anti-inflammatory drugs has also been associated with reduced kidney function in preterm newborns (Cataldi et al., 2005; Cuzzolin et al., 2006). The incidence found in newborns with AKI in a developing country was almost 4/1,000 live births and almost 35/1,000 newborns admitted to the peadiatric unit (Aggarwal et al., 2005).

Some causes of AKI, such as Rapidly Progressive glomerulonephritis (RPGN), may present as AKI but rapidly evolve into chronic renal disease. Several renal diseases, such as the Henoch– Schönlein purpura, hemolytic–uremic syndrome (HUS), and obstructive uropathy with associated renal dysplasia, may present as AKI with significant improvement of renal function to near-normal levels, but the kidney function of the child may slowly deteriorate, leading to renal failure several months or years later. Children with AKI due to hypoxic insults, acute glomerulonephritis, HUS and other causes are more likely to demonstrate oliguria (urine output less than 500 ml/24 h in older children or less than 1 ml/kg per hour in younger children and infants). Children with nephrotoxicity, acute interstitial nephritis, contrast nephropathy, and nephrotoxic renal insults including drugs such as aminoglycoside are likely to have AKI with normal urine output.

The prognosis of AKI is highly dependent on the underlying cause of the injury. Children who have AKI as a component of multisystem failure have a much higher mortality rate than children with intrinsic renal disease such as HUS and RPGN Recovery from intrinsic renal disease is also highly dependent on the underlying etiology of the AKI. Children with aminoglycoside nephrotoxicity and ATN typically recover normal renal function; however, despite in the past it being thought that such patients are at low risk for late complications, it has been shown that chronic kidney disease can evolve from AKI (Askenazi et al., 2006; Andreoli, 2006; Basile et al., 2001).

It is accepted that the concentration of serum creatinine is a delayed and insensitive measure of decreased kidney function following AKI. Other markers under biological investigation include changes in cystatin C and plasma neutrophil gelatinase associated lipocalin (NGAL) levels, and urinary changes in interleukin- 18 (IL-18), NGAL, and kidney injury molecule-1 (KIM-1) (Devarajan, 2007).

## 3. RIFLE Criteria for Staging of AKI

The definition and staging of AKI has been recently standardized using the RIFLE classification proposed by the Acute Dialysis Quality Initiative Group (Bellomo et al., 2004). The RIFLE acronym stands for risk, injury, failure, loss, and end-stage kidney disease. RIFLE defines three increasing grades of severity of AKI (risk, injury, failure) based on a relative increase of serum creatinine. Also, two outcome criteria (loss and end-stage renal disease) are defined. In the first three categories, the RIFLE criteria were developed in order to standardize the definition of AKI by stratifying patients based on the changes in serum creatinine and/or urine output. Temporary loss and permanent end-stage renal disease define two clinical outcome categories based on the length of renal replacement therapy (RRT) after the initiating insult. Despite being a topic of scientific debate (Joannidis, 2007), the RIFLE criteria have been an extremely important development in critically ill adult patients (Hoste et al., 2008).

## 4. Management of AKI

Volume status management in critically ill patients with acute kidney injury is difficult as it is often accompanied by anuria or oligouria as well as total body fluid overload leading to tissue edema. As such, AKI increases the risk of mortality and often occurs in the setting of sepsis or other forms of shock (Bagshaw et al., 2008). While the early goal-directed therapy study showed the benefit of adequate volume repletion in critically ill patients with septic shock (Rivers et al., 2001), there are detrimental effects associated with salt and water overload which can result from resuscitation with colloids and crystalloids. These include difficult wound healing and worsening of lung function (Schrier, 2010; Brandstrup et al., 2003).

Treatment of AKI is subjected to risk stratification and ongoing damage control measures, such as patients with sepsis, exposure to nephrotoxic agents, ischemia, bloody diarrhea, or volume loss, could be helped by optimizing the fluid administrations, antibiotics possessing least nephrotoxic potential, blood transfusion where hemoglobin is dangerously low, limiting the use of nephrotoxic agents including radio contrast use, while maximize the nutrition.

Good nutritional support has been advocated in AKI, since these patients are generally in a developmental growth phase requiring adequate nutrition than adults. Recent data suggests that protein supplementation in critically ill-children of the order of 2-3 g/kg/day for children aged 0-2 years and 1.5-2.0 g/kg/day for children aged 2-13 years (Mehta & Compher, 2009).

## 5. Dopamine Infusion

By using low (renal) dose dopamine in children, AKI outcome remained same (Andreoli, 2009). Another dopamine agonist fenoldopam did not show any promising result in treating AKI although fenoldopam given from 0.05 to 0.1 μg/kg/min was shown to improve creatinine values in 100 adults matched for severity of illness (Brienza et al., 2006).

## 6. Type of Fluid Administration

In adults SOAP and SAFE studies did not show any superiority of crystalloid over colloid administration or vice-versa.(Finfer et al., 2004; Vincent et al., 2006) However, similar studies in children are yet to come.

## 7. Fluid Administration

In a retrospective study of 116 children by (the Prospective Pediatric CRRT Registry Group (ppCRRT), observed, fluid administration to be independently associated with mortality in children started on CRRT, (Goldstein et al., 2005) necessitating the patient specific proper dose of preload fluid administration.

## 8. Diuretics

Converting oliguric AKI into nonoliguric AKI by use of diuretics did not improve the outcome (Swärd et al., 2004). There have been no prospective studies on the use of diuretics in AKI.

To conclude, acute kidney injury remains a complex disorder with an apparent differentiation in pathology between septic and nonseptic forms of the disease. Having common definitions and diagnostic tools aids in the research and, consequently, the management of AKI. With the advent of technology and modern techniques, more research into using these instruments with caution should be carried out (Shah, 2014). Although more studies are still required, progress in this area has been steady over the last decade, with purposeful international collaboration.
